# Rapid Clinical Resolution and Differential Diagnosis of a Neurological Case of Feline Infectious Peritonitis (FIP) Using GS-441524

**DOI:** 10.3390/pathogens14050424

**Published:** 2025-04-27

**Authors:** Amy Huynh, Pamela Moraguez, Logan M. Watkins, Jonathan H. Wood, Ximena A. Olarte-Castillo, Gary R. Whittaker

**Affiliations:** 1 Department of Clinical Sciences, College of Veterinary Medicine, Cornell University, Ithaca, NY 14853, USAjhw29@cornell.edu (J.H.W.); 2 Department of Microbiology & Immunology, College of Veterinary Medicine, Cornell University, Ithaca, NY 14853, USA; pamoraguez@gmail.com (P.M.); xao2@cornell.edu (X.A.O.-C.); 3 Department of Public & Ecosystem Health, College of Veterinary Medicine, Cornell University, Ithaca, NY 14853, USA; lmw255@cornell.edu; 4 Feline Health Center, College of Veterinary Medicine, Cornell University, Ithaca, NY 14853, USA

**Keywords:** feline coronavirus, nanopore sequencing, accurate diagnosis, GS-442524

## Abstract

Case summary: A 2-year-old male neutered domestic shorthair cat was presented with a progressive history of tetraparesis, ataxia, and inappetence over 4 days. A physical exam revealed mucopurulent nasal discharge and stertor. A neurologic exam revealed a multifocal neurolocalization. The cat was non-ambulatory tetraparetic and developed seizures while in hospital. Hematologic assessment revealed anemia, hypoalbuminemia and hyperglobulinemia. Magnetic resonance imaging (MRI) of the brain revealed multifocal meningeal contrast enhancement in the brainstem and cervical spine, as well as mandibular and retropharyngeal lymphadenopathy. Cerebrospinal fluid revealed marked neutrophilic pleocytosis; no infectious organisms were seen. *Toxoplasma* IgG/IgM and *Cryptococcus* antigen latex agglutination were negative. Mandibular and abdominal lymph nodes were aspirated, and cytology revealed mixed inflammation. The cat was suspected to have feline infectious peritonitis, and to aid in clinical diagnosis he was enrolled in research study—with targeted Nanopore-based sequencing specifically identifying and characterizing FCoV-1 RNA in spinal fluid and anal swab, but not in urine. The cat was treated with anticonvulsants (phenobarbital and levetiracetam), an antibiotic (ampicillin/clavulanic acid), and GS-441524. Neurologic signs did not improve on an antibiotic alone but improved significantly after two subcutaneous injections of GS-441524. The cat received an 84-day course of GS-441524 and, at the time of manuscript preparation (over 12 months after diagnosis), remains ambulatory and seizure-free without recurrence of neurologic signs and no detectable viral shedding in feces.

## 1. Relevance and Novel Information

This report demonstrates the rapid resolution of a case of feline infectious peritonitis (FIP) case using a relatively low dose of the antiviral drug GS-441524 (10 mg/kg subcutaneously once daily for 3 weeks, then 10 mg/kg orally once daily for a total 12-week treatment course). This report also suggests an up-to-date method for detecting feline coronavirus RNA, with a Nanopore-based sequencing approach having the potential to be used as a rapid clinical test for an accurate and timely diagnosis of FIP, including in the central nervous system.

## 2. Introduction

Feline infectious peritonitis (FIP) is a deadly infectious disease that affects cats. FIP is caused by feline coronavirus (FCoV), a highly prevalent alphacoronavirus among domestic cats. Without treatment, up to 90% mortality has been reported in kittens [1]. However, recent evidence shows that treatment with antivirals is an effective approach to managing this disease. One of these antivirals is GS-441524, a nucleoside analog metabolite of Remdesivir^®^ that targets the RNA-dependent RNA-polymerase (RdRp, or Nsp12) gene of coronaviruses, stalling viral RNA synthesis [2]. Studies on GS-441524 for treating FIP have demonstrated varying success rates. Early studies using injectable GS-441524 showed improved clinical signs in 68% to 85% of treated cats, with relapses in up to 32% of cats, even after multiple treatment periods [3,4]. However, recent studies using oral GS-441524 have shown complete remission in 100% of tested cats [5,6]. Notably, a shorter treatment time of six weeks, instead of the recommended twelve weeks, achieved the same treatment efficiency [6]. Assessing the effectiveness of short treatments is essential for lowering treatment costs and minimizing the risk of developing antiviral resistance.

FCoV is categorized into two biotypes: a typically non-pathogenic feline enteric coronavirus (FECV), and a highly pathogenic, macrophage-tropic form, feline infectious peritonitis virus (FIPV) [7,8]. FIPV causes a systemic infection and leads to FIP. FCoV is also divided into two serotypes or genotypes: FCoV type 1 (FCoV-1) and FCoV type 2 (FCoV-2). FCoV-2 arises through homologous recombination between FCoV-1 and Canine Coronavirus type 2 (CCoV-2), another closely related alphacoronavirus [9]. Both FCoV-1 and FCoV-2 can cause FIP, but FCoV-1 is more prevalent worldwide [10]. These genotypes possess distinct spike (S) proteins, resulting in variations in their antigenic properties and interactions with the host during cell entry. Both FCoV-1 and FCoV-2 have a cleavage site in the S2 domain of S (S2′ cleavage site), but FCoV-1 has a unique furin cleavage site (FCS) located in spike domain D at the interface of the S1 (receptor binding) and S2 (fusion) domains of S (also referred to as the S1/S2 cleavage site) [11]. Cleavage by host proteases at this site is a critical step for promoting membrane fusion activation [12,13]. Non-pathogenic forms of FCoV-1 (FECV) contain an FCS core consensus motif of -SRRSRRS- (in which S is serine and R is arginine). In contrast, highly pathogenic variants of FCoV-1 (FIPV) found in clinically confirmed cases of FIP typically include one or more mutations in the FCS motif that are believed to modulate viral tropism and spread. Mutations in the S2′ cleavage site have also been associated with some cases of FIP [14]. A mutation in a residue upstream of S2′, M1058L, has also been linked to highly pathogenic FCoV [15], although it is more likely associated with systemic spread rather than FIP per se [16,17]. Statistical comparison of selection intensities between the FECV and FIPV phenotypes available in genomic databases confirmed that a residue within the S1/S2 cleavage site and residue ‘1058’ are associated with FIPV [18]. In this study, no association between mutations in other genes and FIPV was detected, including those genes previously thought to be involved in the pathogenicity (3a, b, c, 7a, b) [18].

Consequently, targeting and sequencing the S1/S2 cleavage site and residue ‘1058’ within the S gene provides fundamental genetic information to diagnose FIP and guide appropriate and timely antiviral treatment. For this purpose, the next-generation sequencing platform from Oxford Nanopore Technologies (ONT) offers many advantages. For example, ONT provides the capability of sequencing long reads, enabling the sequencing of both the S1/S2 region and the residue ‘1058’ in a single PCR of approximately 1200 bp. Similarly, ONT provides real-time sequencing, short library preparation times (ranging from 10 to 200 min) [19], and a portable sequencing device, the MinION (ONT, Oxford, UK), enabling rapid sequencing that can be performed in a clinical setting for swift diagnostics [20]. Although ONT has been widely used for the study of SARS-CoV-2 in domestic cats, its implementation for the study of FCoV is lacking. For example, several recent studies on the epidemiology of FCoV have used Sanger sequencing, which only allows the targeting of small genomic regions [21,22,23]. Additionally, it may require sending samples to a central sequencing facility [13,24], which adds extra time before results are available. In this study, we implement ONT sequencing using the portable MinION (ONT, Oxford, UK) and the small flow cell, the Flongle. We target and sequence the S1/S2 and S2′ cleavage sites and residue ‘1058’ simultaneously in feces and cerebrospinal fluid from a cat showing neurological signs. By sequencing a single PCR, it was possible to analyze the sequencing data using user-friendly programs to obtain rapid and accurate sequencing results. After diagnosis, the cat received GS-441524 and showed rapid improvement after two subcutaneous injections of 10 mg/kg once daily. The cat received a 12-week course of GS-441524 and showed complete resolution of clinical signs. Long-term follow-up revealed that the cat had minimal neurologic signs and was no longer shedding FCoV-1 in its feces.

## 3. Case Description

***Presentation:*** A 2-year-old male neutered domestic shorthair cat (ID#352662) was presented following a 4-day history of tetraparesis, ataxia, and hyporexia. He had a history of an ear infection when he was 1 year old that resolved with oral antibiotics. Post successful treatment, he maintained a residual right head tilt. He was an otherwise healthy indoor cat, had received routine vaccinations, and had no other major medical history.

A general physical examination revealed mild serous ocular, scant mucopurulent nasal discharge, stertor, and persistent tachypnea with increased bronchovesicular sound in all quadrants. A neurologic exam revealed a right head tilt and non-ambulatory tetraparesis. He had absent menace response in both eyes, absent placing and hopping in both thoracic limbs, and reduced hopping and placing in both pelvic limbs. The rest of the neurologic exam was normal. Based on the seizure and exam findings, the patient’s neurolocalization was multifocal: forebrain, central vestibular, and C1-C5 myelopathy. Primary differential diagnoses were infectious (e.g., *Toxoplasma gondii*, *Cryptococcus neoformans*), infectious–inflammatory (e.g., FIP), and neoplasia (e.g., lymphoma), among other less likely causes of this constellation of clinical signs. The cat had a generalized seizure that was treated with a single dose of midazolam (Hospira) 0.3 mg/kg intravenously (IV) and started on levetiracetam (Auromedics) 30 mg/kg IV every 8 h.

A complete blood count revealed normocytic normochromic non-regenerative anemia (HCT 28%, MCV 398 fL, MCHC 31 g/dL, absolute reticulocytes 12,400/ μLuL), inflammatory leukogram with left shift and mild toxic changes (segmented neutrophils 20,800/ μL, band neutrophils 500/ μL), concurrent stress leukogram (lymphocyte 500/ μL, monocytes 2000/ μL), and mild thrombocytopenia (161,000/ μL). Biochemistry revealed mild hypoalbuminemia (2.5 g/dL), severe hyperglobulinemia (6.8 g/dL) with an albumin-to-globulin ratio of 0.4, mildly elevated AST (61 U/L), and moderate hypercholesterolemia (149 mg/dL). Thoracic radiographs revealed a moderate diffuse bronchial pattern and sternal lymphadenopathy. Further workup of the pulmonary changes was recommended and declined by the owner to prioritize the neurologic workup. The patient was placed under general anesthesia and magnetic resonance imaging (MRI) of the brain was performed. The MRI (1.5 T, Siemens Skyra, Malvern, Pennsylvania) revealed multifocal meningeal contrast enhancement of the brainstem and cervical spine as well as mandibular and retropharyngeal lymphadenopathy. Cerebrospinal fluid (CSF) was acquired at the atlantooccipital space. CSF analysis revealed marked neutrophilic pleocytosis with a total nucleated cell count of 583 cells/ μL, red blood cells of 8 cells/ μL, and total protein of 235 mg/dL. No infectious organisms were seen. CSF culture and sensitivity was not performed.

Fine needle aspirates (FNAs) of the left mandibular lymph node were performed, and cytology revealed mixed inflammation and reactive lymphoid tissue with a moderate amount of contracted purple material that was occasionally surrounded by clear space, concerning for *Cryptococcus* versus necrotic debris. *Cryptococcus neoformans* antigen latex agglutination and *Toxoplasma gondii* IgG/IgM antibodies were investigated on serum and were negative. Abdominal ultrasound revealed moderate lymphadenopathy of the mesenteric (jejunal, ileocolic), pancreaticoduodenal, and right medial iliac lymph nodes. Innumerable small hypoechoic splenic nodules with normal splenic size and a mass in the right ventral aspect of the liver that distorted the hepatic margin were noted. Both kidneys had poor corticomedullary distinction. FNAs of the jejunal lymph nodes revealed mild non-degenerative neutrophilic inflammation. FNAs of the liver mass revealed marked pyogranulomatous inflammation and hepatocytes with mild atypia and mild vacuolar change.

***Molecular Diagnosis:*** To aid in clinical management, samples of the cerebrospinal fluid, urine, and anal swab were collected for FCoV-1 screening by PCR followed by next-generation sequencing using the Oxford Nanopore technology, via a research study at Cornell University. The anal swab was placed in DNA/RNA Shield (ZYMO Research) for a week at 4 °C, and then frozen at −20 °C for further use. The CSF and urine samples were immediately processed and then stored frozen at −80 °C. Total RNA was extracted as previously published [25].

FCoV screening was first performed by real-time RT-PCR using previously described primers/probes [26] that target the intergenic region between the Matrix and Nucleocapsid genes of both FCoV-1 and FCoV-2. The iTaq Universal probe one-step kit (BIORAD) was used following the standard protocol [27]. This real-time RT-PCR assay was also used to quantify the FCoV RNA load using an RNA standard of FCoV-1 quantified by ddPCR as previously described [28]. This quantification was performed in duplicates. FCoV RNA was detected in the CSF and anal swab samples, but not in the urine sample (Table 1). While this qPCR allowed confirmation of FCoV infection, it did not provide any information of the genotype (FCoV-1, FCoV-2) or biotype (FECV, FIPV) of the virus present [25]. Therefore, for the two samples that tested positive for FCoV (CSF and anal swab), a partial region of the S gene that includes three regions of predicted relevance for pathogenicity (S1/S2 and S2′ cleavage sites, and the ‘1058’ residue) was further amplified using the novel primers 1263F (5′-TCCTTTCTCACCACAGCAGT-3′) and 1263R (5′-TGCATAGCGAAAGGAACAGC-3′). These primers amplify a region of 1259 bp of the S gene (covering nucleotides 22,298 to 23,556 of the genome of FCoV-1 UU4, accession number FJ938054). We designed our sequencing approach to act as a tool to provide rapid and cost-effective diagnostic support for this case rather than to provide comprehensive genomic analysis. Total RNA was quantified using the Qubit RNA High Sensitivity Reagent (Invitrogen, Carlsbad, CA, USA) in the Qubit 4 fluorometer (Invitrogen). cDNA was synthesized from 100 ng of RNA using the LunaScript^®^ RT SuperMix Kit (NEB, Ipswich, MA, USA) and following the manufacturer’s protocol. The PCR was performed using the Phusion Green Hot Start II High-Fidelity PCR Master Mix (Thermo Fisher Scientific, Waltham, MA, USA) following the 3-step protocol, using 35 cycles, an annealing temperature of 60 °C, and an extension time of 40 s. The final volume of the PCR reaction was 30 μL, and 3 μL of cDNA was used. The resulting amplicons were cleaned using the QIAquick PCR Purification Kit (Qiagen, Germantown, MD, USA), and their concentration was measured using the Qubit dsDNA broad range Assay kit (Invitrogen) in the Qubit 4 fluorometer (Invitrogen); 200 fmol of DNA per sample was barcoded using the Native Barcoding Kit 24 V14 SQK-NBD114.24 (Oxford Nanopore Technologies, ONT, Oxford, UK) following the ‘ligation sequencing amplicons’ protocol. The concentration of the library was measured using the Qubit dsDNA high-sensitivity Assay kit (Invitrogen) in the Qubit 4 fluorometer (Invitrogen). The two samples were sequenced simultaneously in the MinION Mk1B (ONT) using a Flongle Flow Cell R10.4.1 (ONT). The Flongle flow cell was loaded following the protocol for the V14 chemistry and using 50 fmol of the library. The sequencing process was carried out for 1 h using real-time barcoding and fast base calling. After sequencing, high-accuracy basecalling was performed for each barcoded sample using the Dorado v7.1.4 basecaller in the MinKNOW^TM^ software (v. 24.11.10), to generate pass reads with a Phred quality score of at least 20 (Q20+). Quality trimming and removal of amplicon primer sequences were carried out in Geneious Prime 2023.0 (Dotmatics) using the BBDuk Adapter/Quality Trimming Version 38.84 plugin using the Trim Adapters option, where the sequences of the primers were uploaded, along with the Trim Low-Quality option, where the selected minimum quality was 20. In total 37,014 and 46,644 Q20+ sequences were obtained for the CSF and anal swab samples, respectively. The average maximum sequence length obtained was 1301 and 1300 for the CSF and anal swab samples, respectively. The resulting high-quality sequences (QC> 20) were de novo assembled using the SPAdes assemble 3.15.5 [29] in Geneious Prime 2023.0 (Dotmatics) using the default parameters. In total, 23,460 reads with an average of QC of 24.1 were assembled for the CSF sample and 24,560 reads with an average QC of 24.2 were assembled for the anal swab sample. 

The consensus sequences obtained were aligned using the Clustal Omega 1.2.3 algorithm [30,31] and translated to amino acid sequences in Geneious Prime 2023.0 (Dotmatics). The obtained nucleotide sequences were uploaded to GenBank with accession numbers PQ565822 and PQ565823. The nucleotide sequences of the partial region of the S gene (1259 nt) obtained from the anal swab and CSF were 98.3% similar. The sequences of the S1/S2 and S2′ cleavage sites were identical in the two samples and confirmed the presence of FCoV-1 but did not reveal any mutations indicative of a high-pathogenicity virus (Table 1). However, one of the nucleotide differences between the sequences is an A->T mutation in position 1236 of the amplified region (position 23,533 in the genome of FCoV-1 UU4, FJ938054), resulting in an L residue in site ‘1058’ in the sample from the CSF and an M in the anal swab sample. RNA quantification revealed a lower viral RNA load in the CSF (109.94 ± 0.6 copies/µL, Ct 33.95) than in the anal swab (1635.9 ± 168.7 copies/µL, Ct 30.05).

***Treatment:*** In hospital, the cat was maintained in oxygen supplementation due to persistent tachypnea, which resolved after 24 h of supplementation. Ampicillin/sulbactam (Unasyn, West-Ward Pharmaceutical, Exton, PA, USA) was started at 30 mg/kg IV every 8 h due to the inflammatory leukogram with left shift, mucopurulent nasal discharge, and diffuse bronchial pattern, raising concern for possible bacterial upper respiratory tract infection and bacterial pneumonia, and the cat was switched to amoxicillin/clavulanic acid (Clavamox, Zoetis, Parisppany, NJ, USA) at 14 mg/kg by mouth every 12 h prior to discharge. Levetiracetam (Auromedics, Dayton, NY, USA) at 30 mg/kg every 8 h was also started in hospital and he was discharged with levetiracetam (oral suspension, Camber Pharmaceuticals, Piscataway, NJ, USA) at 35 mg/kg every 8 h. He had another generalized seizure in hospital and was also started on phenobarbital (Cameron Pharmaceuticals, Louisville, KY, USA) with a loading dose of 4 mg/kg IV every 6 h for 4 doses, transitioned to 2 mg/kg IV every 12 h, and discharged with phenobarbital (Cornell Pharmacy compounded oral suspension) at 2 mg/kg every 12 h. The cat received antibiotic alone for 3 days and had progressive neurologic worsening characterized by multiple seizures during this period. The owner elected to discharge the cat so that he could receive GS-441524 injections. The GS-441524 was sourced by the owner as at the time this cat was diagnosed, the veterinarian was not legally able to prescribe this medication. At the time of discharge, the cat remained non-ambulatory tetraparetic.

The cat received the first injection of GS-441524 on the evening of discharge. Based on the owner’s communications, the cat received GS-441524 at a dose of 10 mg/kg subcutaneously once daily. After two injections of GS-441524 over the course of two days, the cat’s owner sent a video update showing the cat’s condition had improved significantly and was now ambulatory tetraparetic with proprioceptive ataxia.

***Follow-up:*** The cat received GS-441524 at 10 mg/kg subcutaneously for 3 weeks and was switched over to oral GS-441524 at the same dose for a total of 12 weeks on this medication. The cat had serial follow-up neurologic exams at 2 weeks, 10 weeks, and 36 weeks after diagnosis. Follow-up exams showed steady improvement of neurologic signs, and the cat remained seizure-free and anticonvulsants were discontinued. Fecal samples or anal swabs were collected 7, 15, 17, and 252 days after the initial treatment with GS-441524 (Table 1). FCoV-1 RNA was not detected in any of these samples (Table 1).

The patient was rechecked 2 weeks after his initial discharge. A physical exam revealed resolved ocular and nasal discharge but persistent stertor. The neurologic exam revealed that he was ambulatory without paresis but had persistent proprioceptive ataxia, absent menace in both eyes, and a persistent right head tilt. The patient had no further witnessed seizure activity since discharge. A complete blood count revealed a resolved inflammatory leukogram. Serum biochemistry revealed improved hypoalbuminemia (3.0 g/dL) and hyperglobulinemia (5.1 g/dL) with an albumin-to-globulin ratio of 0.6. His phenobarbital level was 18 µg/mL (therapeutic range: 10–30 µg/mL). Further workup was recommended for the cat’s respiratory signs but the client declined these diagnostics. Fecal sample swabs were collected 7, 15, and 17 days after antiviral treatment for FCoV-1 screening as described above. None of these three samples were positive for FCoV-1 (Table 1). No additional samples of spinal fluid were taken due to no clinical indication to perform this test, given the patient’s improvement and the risk of generalized anesthesia required to obtain further CSF samples. Amoxicillin/clavulanic acid (Clavamox, Zoetis) was discontinued, and the cat was maintained on phenobarbital and levetiracetam at the same doses listed above. The cat was maintained on subcutaneous injection of GS-441524 of 10 mg/kg for 22 days and then transitioned to oral GS-441524 with the same dose thereafter. At the time that the cat was undergoing treatment for FIP, veterinarians were not able to legally prescribe or make recommendations for dosing of GS-441524, and therefore there was no input regarding dosing adjustment for the switch from injectable to oral GS-441524.

At the 10-week recheck exam, the cat continued to be mildly stertorous, and the remainder of the physical exam was normal. The neurologic exam revealed that he continued to be ambulatory without paresis but had persistent mild proprioceptive ataxia, absent menace in both eyes, and a right head tilt. A complete blood count showed new eosinophilia (4100/μL), and biochemistry revealed normalized albumin and globulin levels. Cholesterol levels continued to be low (109 mg/dL). A test for *Dirofilaria immitis* antigen, feline leukemia virus antigen and feline immunodeficiency virus antibody was performed to assess for possible *Dirofilaria immitis* infection as the cause for persistent respiratory signs. The cat was negative for *Dirofilaria immitis* but incidentally found to be positive for feline leukemia virus antigen. This test was also performed prior to presentation to our hospital and was negative in the past. A feline leukemia virus quantitative real-time polymerase chain reaction was performed and consistent with progressive infection (170.58 × 10^6^ copies/mL). The cat may have had a regressive infection that became progressive due to immunosuppression from FIP. Alternatively, the cat may have recently acquired feline leukemia virus and was falsely negative in the first antigen test. The intersection between feline retroviral infection and FIP has been well documented in the past [32], where up to 20% of cats with suspected FIP were coinfected with feline leukemia virus and about 15% had both feline leukemia and feline immunodeficiency virus. It is possible that the recently acquired feline leukemia virus caused immunosuppression that facilitated rapid central nervous system involvement of the low-pathogenic virus identified via Nanopore sequencing. The cat’s MRI was reviewed once again at this time and nasopharyngeal stenosis was noted. Further workup for respiratory signs was once again recommended and declined by the owner. The cat was restarted on amoxicillin/clavulanic acid (Clavamox, Zoetis) at the same dose as previously for an additional 6 weeks. A fecal analysis by zinc sulfate centrifugal flotation identified Giardia, which may be the cause of peripheral eosinophilia. Peripheral eosinophilia has also been described as a common clinicopathologic abnormality for cats during treatment GS-441524 [33], occurring in 50–60% of cases, and resolves spontaneously after discontinuation of the medication. The cat was no longer on levetiracetam as the client had stopped giving this medication after one month. He was continued on phenobarbital at the same dose, started on fenbendazole (oral powder, Merck, Rahway, NJ, USA) at 50 mg/kg once daily for 5 days for giardiasis, and started on a hydrolyzed diet (Purina Pro Plan HA, St. Louis, MO, USA) due to concern for enteropathy given hypocholesterolemia. Oral GS-441524 was continued for a full 12-week course of treatment.

At the 36-week recheck, the cat had persistent mild stertor and the rest of the physical exam was normal. The neurologic exam revealed that he was ambulatory without paresis or ataxia, persistent absent menace in both eyes and persistent right head tilt. A complete blood count and biochemistry were both within normal limits. Conjunctival and rectal swabs were obtained and were negative for FCoV RNA (Table 1). Phenobarbital was tapered off due to no seizures since discharge from the hospital.

The cat remained ambulatory without paresis or ataxia and was seizure-free during the 12-month follow-up period.

## 4. Discussion

FIP is relatively common in young cats with neurologic signs [32]. This disease presents challenges for clinicians, particularly in early diagnosis and establishing effective therapeutic interventions. Clinical management of FIP increasingly involves treating affected cats with antiviral drugs [4,34,35]. Currently, IV and subcutaneous formulations of GS-441524 are not routinely available for veterinary patients, although IV Remdesivir^®^ has been used as an alternative nucleoside analog [35] and remains in use in specific cases. Oral GS-441524 formulations are now routinely available, and their efficacy has been demonstrated in multiple studies [5,36]. Cats exhibiting neurological signs, however, are relatively understudied and often require higher doses and extended treatment durations [4]. Here, we report a case of rapid diagnosis and clinical resolution in a cat with confirmed central nervous system FIP, treated with owner-sourced antiviral GS-441524. We are also describing a novel method for a rapid antemortem diagnosis of FIP.

In this study, we employed Nanopore-based next-generation sequencing to detect FCoV-1 in the cerebrospinal fluid (CSF) of a cat with neurological symptoms. Previous studies from our lab used Sanger-based sequencing [12,37], which posed delays due to the need for centralized processing. For example, the turnaround time for obtaining Sanger sequencing results at Cornell’s Biotechnology Resource Center is approximately 16 to 32 h after the samples have been delivered to the sequencing facility. Here, we demonstrated the use of portable MinION (ONT) sequencing at a clinical facility, offering significant advantages in speed and convenience, allowing for rapid screening for FCoV-1. For example, we were able to obtain sufficient reads (>37,000) for each sample after one hour of sequencing using the small Flongle flow cell to obtain a segment of the S gene (1259 bp long). In comparison, Sanger sequencing typically yields two sequences: one from the forward primer and the other from the reverse primer. More in-depth Sanger sequencing requires cloning the PCR product, which adds extra time to the process. The length supported by Sanger sequencing is also limited (up to 1000 bp, with an expected low quality for the first 40 bp due to the primer binding) [38]. The average maximum length of the sequences obtained in this study was 1301 bp, which allowed us to include three relevant regions that are considered markers of pathogenicity. Longer regions were not targeted because the S1 region of FCoV-1 is highly variable, which complicates the generation of primers, resulting in low sensitivity and hindering the possibility of sequencing the S gene compared to other, more conserved genes [21,39]. Since the assay consists of a single PCR product, it was possible to perform the QC analysis and general sequence analysis using a user-friendly program (Geneious Prime 2023.0), resulting in high-quality data that does not require the use of advanced bioinformatic platforms. Although the ONT platform has been widely used for the study of SARS-CoV-2 in domestic cats [40], it has been only recently used for the study of FCoV [41]. Therefore, our study confirms that this technology can be utilized for the study of FCoV in clinical settings without requiring advanced laboratory equipment or bioinformatics platforms.

Previous studies targeting the S gene have focused on small regions of the S gene, including mostly the ‘1058’ site [13,21]. The PCR method in this study targeted the S1/S2 cleavage site (the “furin cleavage site”) in spike domain D, the S2′ region adjacent to the fusion peptide, and residue “1058” (residue 1046 in reference strain UU4 [42]) of FCoV-1. Interestingly, the sequence data of the S1/S2 cleavage site showed a sequence more indicative of a “low-pathogenicity” virus, i.e., indicated by non-mutated cleavage sites compared to reference ‘FECV’ strains [12], but with mutation M1058L consistent with systemic spread [16]. Additional sequencing may reveal further mutations in other genomic hot spots associated with a “high-pathogenicity” virus (FIPV) as they become identified. Notably, RNA quantification indicated a low FCoV-1 RNA load in the CSF (109.94 ± 0.6 copies/µL, Ct 33.95). This may indicate an early infection stage, whereby the virus had not yet had time to acquire mutations associated with robust macrophage tropism and widespread tissue distribution. Such an understanding of FIP-associated mutations will be critical in future diagnostic schemes. A previous study also detected very low viral loads of FCoV in the CSF of cats with confirmed FIP and displaying various signs [43]. In this study, due to the low viral load, detection of mutations on S was only possible in 10% of the screened samples, all of which had the M1058L mutation. Another study also found low viral loads in CSF in cats showing neurological signs, indicated by relatively high Ct values by qPCR (>26.5) [44]. Nevertheless, this qPCR in CSF was highly specific for detecting FCoV in the central nervous system, confirming an FIP diagnosis. The FCoV-1 detected in the feces and that in the CSF were 98.3% similar. As the targeted region is the S gene, intra-host variation was expected, especially in the S1/S2 region, which is related to pathogenicity. For example, certain mutations in S1/S2 have been reported only in tissues of the central nervous system (i.e., spinal cord, brain), but not in feces of the same cat [45].

While lymph node FNAs were collected in the clinic, they were unavailable for our research study. However, they could potentially be predictive for diagnosis via cytology or molecular methods [46,47] and could serve to validate our CSF sequencing results, offering insights into FCoV-1 evolution in this case and mutational changes associated with FCoV-1 pathogenesis [18]. We did not assess ORF3 or ORF7 due to their limited predictive value in FIP diagnosis. This study underscores the utility of evaluating both “low-path” and “high-path” viral sequences alongside clinical parameters. In a previous study from our lab, we identified mutations in the S1/S2 cleavage site consistent with an “internal mutation” of FCoV-1 [45]; however, this was in a fatal neurological case with virus evolution in macrophage-like cells within the neural tissue but not in other organs.

The cat in this study achieved clinical and diagnostic remission by the time of publication. The patient will continue to be monitored to assess the long-term efficacy of GS-441524 in suppressing FCoV replication and shedding.

One of the limitations of our case report is that we have a single case, and therefore we cannot establish the sensitivity and specificity of our technique. Moreover, the GS-441524 was sourced by the owner rather than from a licensed source due to veterinarians’ inability to prescribe this medication at the time this cat was diagnosed with FIP. Unlicensed products may contain different amounts of GS-441524 than advertised [48] and therefore the cat may have received a higher dose of GS-441524 than the reported 10 mg/kg daily dose. Veterinarians were also unable to directly participate in the recommendation for dosing and duration of treatment due to this limitation. However, recent changes have allowed veterinarians the ability to legally prescribe this medication, and oral GS-441524 is also legally available, allowing for more transparency and cooperation between owners and veterinarians during the treatment of FIP.

## Figures and Tables

**Table 1:** Summary of the samples collected and the result of the FCoV-1 screening.

Sample Collection Date	Sample Type	FCoV-1 Status	Amino Acid Sequence of the S1/S2 Furin Cleavage Site	Amino Acid Sequence of the S2′ Cleavage Site	Amino Acid Residue at Position “1058”
29 August 2023 *	CSF	Positive	SRRSRR|STSESV	KR|S	L
29 August 2023 *	Anal swab	Positive	SRRSRR|STSESV	KR|S	M
29 August 2023 *	Urine	Negative	N/A	N/A	N/A
5 September 2023	Feces	Negative	N/A	N/A	N/A
13 September 2023	Feces	Negative	N/A	N/A	N/A
15 September 2023	Anal swab	Negative	N/A	N/A	N/A
7 May 2024	Anal swab	Negative	N/A	N/A	N/A

* indicates samples that were taken before the treatment with GS-441524.

## Data Availability

The original contributions presented in this study are included in the article. Further inquiries can be directed to the corresponding author.
